# Unfolding Visual Lexical Decision in Time

**DOI:** 10.1371/journal.pone.0035932

**Published:** 2012-04-26

**Authors:** Laura Barca, Giovanni Pezzulo

**Affiliations:** 1 Institute of Cognitive Sciences and Technologies, National Research Council (ISTC-CNR), Rome, Italy; 2 Istituto di Linguistica Computazionale “Antonio Zampolli," National Research Council (ILC-CNR), Pisa, Italy; University of Leicester, United Kingdom

## Abstract

Visual lexical decision is a classical paradigm in psycholinguistics, and numerous studies have assessed the so-called “lexicality effect" (i.e., better performance with lexical than non-lexical stimuli). Far less is known about the dynamics of choice, because many studies measured overall reaction times, which are not informative about underlying processes. To unfold visual lexical decision in (over) time, we measured participants' hand movements toward one of two item alternatives by recording the streaming x,y coordinates of the computer mouse. Participants categorized four kinds of stimuli as “lexical" or “non-lexical:" high and low frequency words, pseudowords, and letter strings. Spatial attraction toward the opposite category was present for low frequency words and pseudowords. Increasing the ambiguity of the stimuli led to greater movement complexity and trajectory attraction to competitors, whereas no such effect was present for high frequency words and letter strings. Results fit well with dynamic models of perceptual decision-making, which describe the process as a competition between alternatives guided by the continuous accumulation of evidence. More broadly, our results point to a key role of statistical decision theory in studying linguistic processing in terms of dynamic and non-modular mechanisms.

## Introduction

The lexicality effect (i.e. faster and more accurate responses in processing words than nonwords) has been assessed in numerous psycholinguistic studies. In reading aloud Italian stimuli words are named faster than nonwords, regardless of their frequency (high or low) or list composition (pure vs. mixed blocks) [1], or the readers' expertise [2]. In lexical decision tasks, when participants are required to briefly categorize items presented as words or nonwords, the Lexicality effect should be attenuated if the comparison is made between extremely lexical items (i.e., high frequency words) and extremely nonlexical items (i.e., strings of consonants), because the discrimination between stimuli does not require in-depth analysis but can be based on the visual processing of items [3].

Although stimuli are ultimately categorized as either lexical or nonlexical, the underlying lexical decision is not necessarily a discrete process. In fact, we argue that it would be better described in terms of a dynamic competition between candidate alternatives (in our case, lexicality vs. non-lexicality of the stimulus).

This links lexical decision to state-of-the-art models of decision-making and statistical decision theory. These models describe the choice between two possible stimulus categories as a dynamic process in which partially active alternatives are maintained in parallel and compete over time through the noisy accumulation of relevant information up to a “decision bound" [4]–[7]. Drift-diffusion models [6] describe choice as the continuous accumulation of evidence in favor of the choice alternatives, as encoded in decision variables. In our case, decision variables could included orthographic and lexical evidence. Race models [7] are closely related to drift-diffusion models but argue that evidence is maintained separately for the competing hypotheses. Similarly, Spivey [8] describes choices in terms of a continuous, dynamic competition between attractors (in this case, the two choice alternatives); these attractors “push" and “pull" mouse trajectories in a putative dynamic mental space. An alternative to this (bottom-up) evidence accumulation approach is the *predictive coding* view in which choice is essentially the minimization of the discrepancies between top-down predictions (e.g., lexical predictions) and bottom-up (perceptual) stimuli (see e.g., [9]–[12]). According to this model, the brain maintains a generative model of the sensorium, which encodes (probabilistic) hypotheses concerning the identity of observed stimuli (in this case, words or nonwords) and uses it to continuously generate (lexical) predictions. These predictions are propagated top-down in the processing hierarchy. By matching them against the actually observed stimuli, a prediction error signal is generated, which is propagated bottom-up and serves to revise the hypotheses at the highest level of the hierarchy. The selected hypothesis is the one that (after a sufficient number of iterations) minimizes a certain measure (in Friston's account [10], [11], free energy, or with some approximation, prediction error).

Although all of these models are different, they describe decision-making as a dynamic process in which alternatives compete over time and conflict is solved (in a statistically sound manner) by accumulating evidence in favor of (or against) the alternatives. This dynamic view of decision-making is common to many models of linguistic processing; for example, most models of spoken word recognition emphasize competition between partially active lexical representations [13]–[17]. Here we applied the same logic to lexical decisions and emphasized two important factors that are less studied in these frameworks. First, although most models of dynamic decision-making assume that decisions are made before the action onset (e.g., before the subject presses a button), here we argue that this is not the case and that the dynamic competition continues as the subject responds, and can be revised at later stages (In fact, this is why it can be studied by measuring action kinematics during the response performance; see the discussion). According to Spivey et al. [18], the continuous merging of information necessary to make a lexical decision is reflected in the continuous execution of motor output, “consistent with a nonstop cascaded sharing of information among perception, cognition and action". Second, although most models assume that the source of dynamicity of choice is in the nature of the stimulus (i.e., the fact that spoken words unfold over time), in this study we focus on *internal* sources of dynamicity, which depend on the way evidence is considered and integrated during lexical decision.

To understand how this is possible, two considerations are in order. First, it is important to consider how “decision variables" are selected, that is, what are the relevant dimensions along which evidence is accumulated for or against the lexicality of a stimulus. The psycholinguistic literature suggests that many sources of information, that is, visual, orthographic, phonological, and semantic, are all potentially relevant to the choice [15]. A second important issue is how (perceptual) inference is resolved or how a certain piece of evidence (e.g. the fact that the stimulus includes a certain bigram) is counted as evidence in favor of one of the two alternatives (the stimulus being is lexical vs. non-lexical). The psycholinguistic literature suggests that perceptual processing of linguistic stimuli is a stochastic process and requires access to (and comparison with) stored memory representations (represented as trace vectors of feature values in Shiffrin and Steyvers' [17] REM model or maintained in the reciprocal connections of cortical hierarchies in Price and Devlin's [19] interactive account). In the context of a lexical choice, the successful “matching" of stimulus features with memory representations (potentially many kinds, e.g., visual, orthographic, phonological, and semantic) can be considered evidence of the lexicality of the stimulus.

In principle, the perceptual-memory process described above could provide the (semi) continuous in-flow of evidence required for the dynamic models of decision-making to work. One complication, however, is that this process is non-stationary, and evidence in favor of each of the alternatives can be stronger or weaker at different intervals (see Tsetsos et al. [20] for a recent discussion on non-stationarity in dynamic models of decision-making). There are two reasons for this: first, perceptual inference (and memory retrieval) of different kinds of information, for example, visual vs. semantic, could require more or less time and therefore evidence would be collected at different speeds for these elements; second, consistent with several models of lexical processing, we assume that the strength of the memory trace significantly modulates the process because it is quicker for more familiar stimuli (e.g., high frequency words) than less familiar stimuli (e.g., low frequency words).

If one considers jointly the importance of different decision variables and their non-stationarity, it emerges that lexical decision is a multifaceted decision-making process in which the initial choice can be revised when novel information (e.g., semantic information) becomes available. We hypothesized that it consists of a dynamic process of accumulation (and comparison) of evidence for or against the lexicality of stimuli. Furthermore, we hypothesized that the lexicality dimension is not an all-or-nothing characteristic of stimuli. At least in the context of lexical decisions, linguistic and pseudo-linguistic stimuli could be ordered along a lexicality dimension or a “lexical dimension line" (analogously with the “number line" [21]), which functions as a continuum between highly lexical items (i.e. words with high frequency values), weak lexical items (i.e. words with low frequency values), weak nonlexical items (i.e. legal pseudowords) and highly nonlexical items (i.e., strings of letters).

If our hypotheses are correct, the lexicality dimension of stimuli (words and pseudo-words) should affect the unfolding of the lexical decision process in time. Continuous measures of processing are more informative about the dynamics of choice than reaction time experiments. Thus, to test our hypotheses we measured participants' kinematics (i.e., mouse movements) during a lexical decision task involving the four kinds of stimuli described. In our experimental set-up, participants performed the lexical decision task by moving the mouse to indicate their response. Using the MouseTracker apparatus [22], we tracked continuous hand movement responses during a visual-lexical decision task to observe the graded effects of competing items attracting the trajectory of the mouse also during trials in which the categorization was correctly executed. This technique has been successfully adopted in psycholinguistic studies, and complements other techniques such as the measurement of reaction times and saccadic eye movements. As it tracks continuous reaching movements, the technique allows studying the dynamics of choice between multiple competing hypotheses during response and can reveal graded processing and uncertainty throughout the response. For instance, using this technique Spivey et al. [9] reported the partial activation of multiple lexical representations cascading to later stages of processing during spoken word recognition. In this study, we aimed to further corroborate the existence of this dynamicity in language processes by demonstrating that the lexicality of stimuli modulates hand movements in a visual lexical decision task.

Specifically, in this study we expected no interference from competitors in the processing of *high frequency words* and *letters strings*. Therefore, hand movements/mouse trajectories should not be pulled toward the opposite category and response times should be fast and accurate. In the case of *low frequency words*, the mouse trajectory might be pulled to the nonlexical item category due to competition; in fact, responses might be slower than words and letter strings but faster than pseudowords, reflecting the advantage of (weak) lexical representations. There might be more errors than high frequency words and strings of letters. For *pseudoword* trajectories, there should be a relevant attraction to the lexical category, with an effect on both reaction time and accuracy rate (i.e., slower response and more errors than to all other stimuli).

One advantage of adopting the MouseTracker apparatus is that it allows gathering several measures of participants' responses not restricted to overall timing and accuracy of the response. Measures such as curvature areas, switches of movement direction or movement complexity might be relevant in clarifying the dynamics of the lexical decision/revision processes underlying the more ambiguous stimuli (e.g., Pseudowords or Low Frequency words). It might be possible to track other differences by analyzing when the participants initiate their hand movements (i.e. Initiation Time, which measures the time from when the mouse becomes active and the participants first move it) and whether movement direction was ever drastically modified along the x-axes (i.e., Maximum Deviation time). Another measure, the Area Under the Curve of a trajectory (AUC), is calculated as the geometric area between the actual trajectory and the idealized trajectory (straight line from the start to the response button). If response alternatives simultaneously attract participants' mouse trajectories (relative to only one), this might manifest as less smooth, more complex trajectories. Fluctuations in the vacillation of the hands along the horizontal axes are indexed by measuring x-flips, which is the number of reversed directions along the horizontal axis. However, these measures could reflect very different processes. Difference in Initiation Times might reflect some impulsive behavior in responding, which might be driven by activation of the lexical representation. And the x-flip might pertain more to a revision stage of the decision process, when top-down phonological/semantics representations allow revising and correcting the decision.

## Methods

### Ethics Statement

The procedure was approved by the Institute of Cognitive Sciences and Technologies of the National Research Council, ISTC-CNR of Rome. Informed consent was obtained from all participants.

### Participants

The study included 22 highly educated (university students or young researchers) native speakers of Italian, whose ages ranged from 20 to 35 years. All were right-handed with normal or corrected to normal vision. )

### Materials and stimuli

Experimental stimuli consisted of a list of 96 lexical and non-lexical items. The lexical items were 48 singular Italian nouns taken from Barca, Burani and Arduino's [23] database. All were five letters long, morphologically simple (i.e. neither derived nor compounds) and unambiguous as to grammatical category and meaning. Written frequency was manipulated: high frequency words had a mean value of 536.9 (ranging from 151 to 1370) and low frequency words had a mean value of 6.9 (ranging from 2 to 11). Written frequency it's a measure of “adult written word frequency" taken from a frequency count based on a written corpus that comprises 3.798.275 lexical occurrences (CoLFIS; http://www.istc.cnr.it/material/database/colfis/index_eng.shtml). All but two words were regularly stressed on the penultimate syllable. The two exceptions were stressed on the antepenultimate syllable: “EPOCA" (/epoch/) a high frequency word, and “ELICA" (/propeller/), a low frequency word.

Stimuli also varied for rule contextuality. Therefore, half of the list included non-contextual graphemes (“LATTE", /milk/, made up of letters with a one-to-one mapping between grapheme and phoneme) and half of contextual graphemes (“CERVO", /deer/, made up of letters such as /c/ or /g/ whose pronunciation depends on the letters that follow them) (see [24], [25]). Table 1 visualizes the psycholinguistic characteristics of the stimuli).

**Table 1:** Psycholinguistic characteristics of the word stimuli, mean values and standard deviation (in parentheses).

	AoA	Fam	Ima	OrtNeigh	BigrFreq
**High Frequency**	2.83 (1.13)	6.62 (0.42)	5.06 (1.43)	1.88 (1.70)	10.84 (0.41)
**Low Frequency**	3.78 (0.94)	5.62 (0.84)	5.13 (0.90)	1.96 (1.53)	10.49 (0.63)

Legend: AoA = Age of Acquisition; Fam = Familiarity; Ima = Imageability; OrtNeigh = Orthographic Neighbors; BigrFreq = Bigram Frequency.

High frequency words tend to be acquired earlier (p<.05) and are more familiar (p<.005) than low frequency words, but were similarly imageable nouns, with small number of orthographically similar words, and made up of similarly frequent bigrams (all p = ns.). Non-lexical items included 24 pseudowords and 24 strings of letters. Pseudowords were created by changing two or more letters of real low-frequency words (not included in the list), so that they were pronounceable and orthographically similar to the lexical stimuli. They varied for grapheme-phoneme contextuality, so there were equal numbers of pseudowords such as “GHEBO" and “NUPIA".

Letters strings were created by randomly assembling the letters of the Italian alphabet (thus, the letters “w" and “y" were not used). To improve stimulus variation in the experimental list, half of the stimuli were strings of consonants (“BTFPR") and half were strings of vowels (“IEIOU").

Thus, the experimental stimuli could be arranged along a “lexicality dimension line", ranging from highly lexical items with reach lexical representations to one extreme. At the opposite pole were strings of letters that had no representations in the lexicon and could not be assembled into orthographic/phonological sequences, and did not resemble any lexical items. More ambiguous items, such as low frequency words and pseudowords, which we expected to be attracted to their relative competing category, were placed in between.

### Procedure

To begin each trial, participants clicked on the /START/ button located at the bottom-center of the PC screen. Then a fixation cross appeared at the center of the screen, which was replaced by an experimental stimulus after 300 ms. The stimuli remained on the screen for 500 ms. Participants had to respond within 2000 ms, otherwise a /TIME OUT/ message appeared. Stimuli were presented in ARIAL font, upper case black print on a white background. The use of upper case letters allowed controlling for variation in visual features of letters and words, ensuring that letters in the stimuli are always equally spaced and stimuli have the same physical length. Participants were instructed to use the mouse to move the cursor to the appropriate response (i.e., top-left button for lexical stimuli, top-right button for non lexical stimuli) and to click it to indicate their response. The correspondence between stimulus type and button was varied across participants.

Categorization errors and reaction times (i.e. from when participants pressed /START/ until they reached and pressed the response button) were recorded automatically. In the case of errors, a feedback message (red cross) appeared after the response.

While the participants responded, the x and y coordinates of the mouse trajectories were recorded (sampling rate of approximately 70 Hz) using MouseTracker. This package was used to record, process, and analyze mouse movements [21]. Before the experimental data were acquired, the participants performed a practice session of 12 items (6 lexical stimuli and 6 non lexical stimuli) to become familiar with the procedure. The 96 experimental stimuli were presented in two blocks of 48 items each. The order of stimuli within blocks and the order of block presentation were randomized. Half of the participants categorized lexical stimuli using the left button and nonlexical stimuli on using the right button, and the other half did the opposite.

## Results

### Data Processing

Several steps (adapted from previous studies that used MouseTracker software [22]) were performed to allow comparison between trajectories. First, trajectories were rescaled into a standard coordinate space. The top-left corner of the screen corresponded to “−1, 1.5" and the bottom-right corner to “1, 0". In our two-choice design, this left the start location of the mouse (the bottom-center) with coordinates “0, 0". Thus, this standard space represented a 2×1.5 rectangle, which retains the aspect ratio of most computer screens. Then the duration of the trajectory movements were normalized by re-sampling the time vector into 101 time-steps using linear interpolation to allow averaging across multiple trials.

Responses exceeding the 2000 ms deadline, which accounted for 5.65% of the total data, were discarded from the analysis, as were incorrect responses (i.e. when the subject selected the inappropriate stimulus category), which accounted for 3.2% of the total data. Thus, a total of 9% of the responses were discarded from subsequent analysis of the reaction times parameter and trajectories.

We applied Linear Mixed-Effects Modelling (LMMs) to assess the impact of Lexicality on the response variables. LMMs allows estimating the magnitude of variation deriving from the pool of subjects and items and overcomes the limitation of conducting independent F1 and F2 analyses [26], [27]. Subjects and Items were considered Random-effects factors and Stimulus category, a Fixed-effects factor. As the Stimulus category has four levels (i.e., High and Low Frequency words, Pseudowords and Letters Strings), High Frequency words, which are the stimuli with the richest lexical representations, were considered as the “default" level for comparison.

Separate models were run for the different dependent variables (i.e., accuracy rate, initiation time, etc.). Analyses were run with the lm4 package for R [28], where p values were estimated using the Markov chain Monte Carlo simulations [26].

### Accuracy, Initiation Time and Lexical Decision time

**Figure 1:**
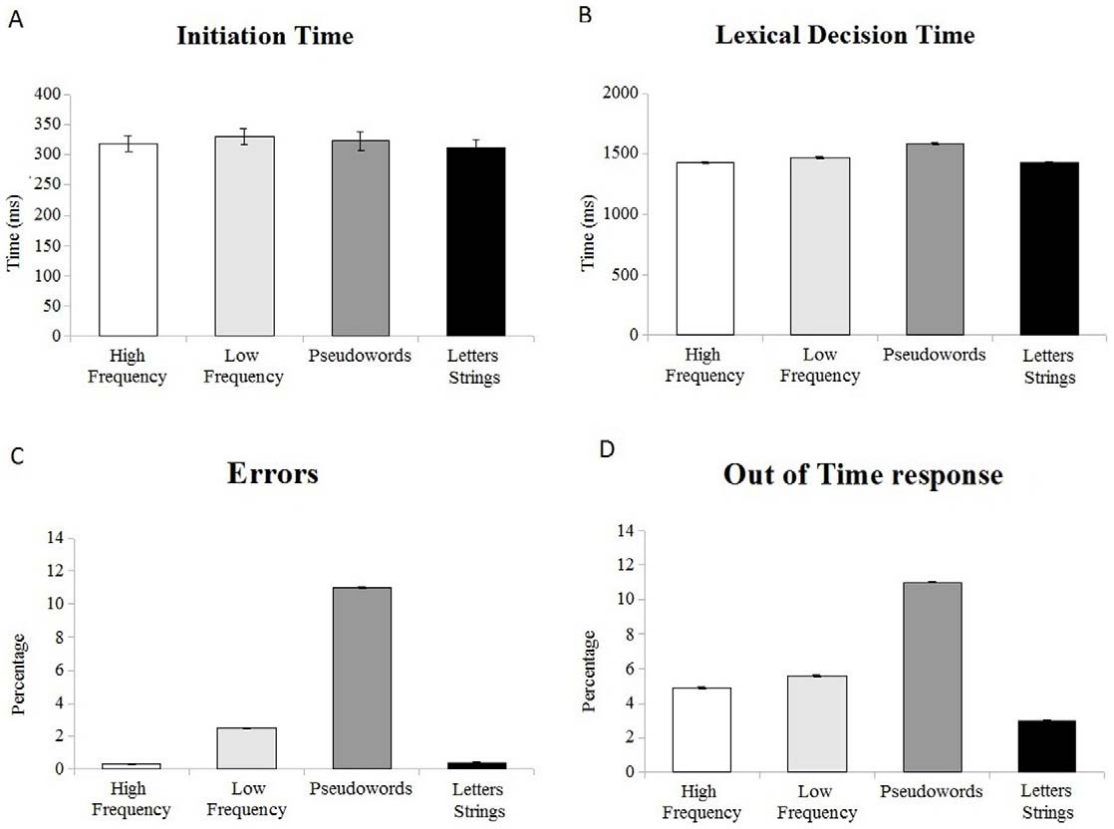
Participants' performance on a visual lexical decision task, in which responses were modulated by the lexicality of the stimuli in accuracy parameters and overall lexical decision time. Error bars depict Standard Error of the mean. a) Average time of movement initiation; b) Average lexical decision reaction time; c) Percentage of categorization errors; d) Percentage of responses after the deadline.

Mean accuracy rates, initiation times (when mouse movements started) and total response time were computed for each participant by averaging trials across each condition (see Figure 1).

Inspection of the bar plots indicates that when participants initiated their movement response there was no difference between stimuli. Likewise, there was not much difference in total reaction time. Pseudowords were the stimulus category with the higher number of errors and with similar proportions of errors and “time out" responses. Participants were more accurate in the other stimulus category (particularly on highly frequent words and letter strings).

In the mixed-effects models of *Initiation Time*, no predictors were significant, indicating that the lexicality variation did not significantly modulate the participants' timing when they started to move the mouse, but affected overall reaction time. Results of the *Lexical Decision Time* analysis showed that Low Frequency words and Pseudowords were slower than High Frequency words (β _LowFreq_ = 43, tvalue = 4.2, β _Pseudowords_ = 158, tvalue = 12), and that participants' speed on High Frequency words and Letters Strings was comparable (β _LetterStrings_ = 1.3, tvalue<1). Directly testing the contrast between Pseudowords and other stimuli showed that Pseudowords were significantly slower than Letters Strings (β _Pseudowords_ = 157, tvalue = 11.8) and Low Frequency words (β _Pseudowords_ = 114, tvalue = 7.4)

In the model with *Accuracy* as the Dependent Variable, a Logistic LMMs for binomial distribution was used. The analysis showed that participants made more errors on Low Frequency words (β _LowFreq_ = 3.9, Zvalue = 3.8 pr<.005) and Pseudowords (β _Pseudowords_ = 4.9, Zvalue = 5.5, pr<.001) than High Frequency words, as indicated by positive beta coefficients. The differences between Low Frequency and Pseudowords (β _Pseudowords_ = 1.01, Zvalue = 2.5, pr<.05) and between Letters Strings and Pseudowords (β _Pseudowords_ = 3.69, Zvalue = 3.4, pr<.001) were also significant, with more categorization errors for Pseudowords. No difference emerged between High frequency words and Letters Strings (β _LetterStrings_ = −9.1, Zvalue = −.49, pr>.1). The “*Out of time*" response analysis also showed a higher proportion of “Out of Time" trials for Pseudowords than highly frequent words (β _Pseudowords_ = .98, Zvalue = 3.3, pr<.005), with no other effects for this measure.

Results suggest that Stimulus lexicality has a role in accuracy rate and temporal dimensions as overall response time. Nevertheless, the distribution of response time might have undergone a ‘shrinking’ because we set the response deadline at 2000 msec (see previous section for details of experimental procedure). If this is true, pseudoword RTs should appear faster because their longer times have been eliminated as outliers exceeding the time deadline, and might suffer more than other categories because they have a higher proportion of “Out of Time" trials. To explore this possibility, the “Out of Time" trials were included in reaction times and submitted to mixed-effects modeling. Results of Lexical Decision time were confirmed, with parameters for Low frequency words (β_Low frequency_ = 51, tvalue = 4.02) and Pseudowords (β _Pseudowords_ = 195, tvalue = 13.3) significantly different from highly frequent stimuli, and Pseudowords slower than Low Frequency words (β _Pseudowords_ = 145, tvalue = 8.7) and Letter Strings (β _Pseudowords_ = 208, tvalue = 13.8). Thus Pseudowords and Low Frequency stimuli were slower than rich lexical items, with a mean reaction time difference for Pseudowords and High Frequency words about three times larger than the difference between Low and High frequency words.

### Trajectory analysis

#### Spatial attraction

**Figure 2:**
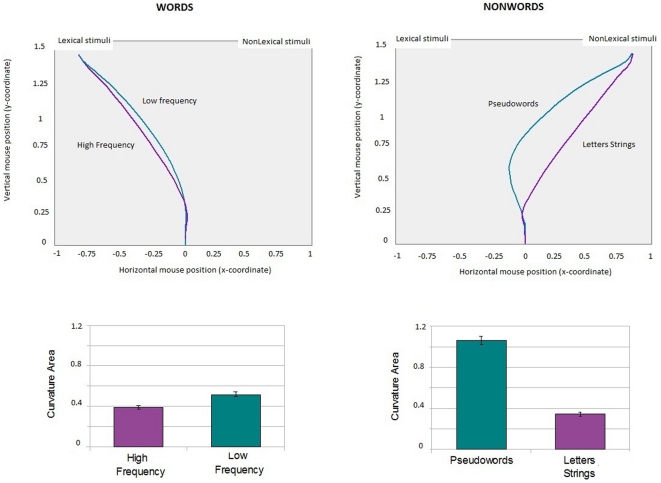
Real-time mouse trajectories. A) Trajectories for High and Low Frequency words. Correct category is on the left and the opposite category is on the right. Trajectories for LF words showed attraction to the ‘nonlexical’ response alternative, which was statistically significant as indexed by AUC (bar plot). B) Trajectories for Pseudowords and Letter Strings. The correct category is on the right and the opposite category is on the left. Trajectories for Pseudowords showed attraction to the ‘lexical’ response alternative, which was statistically significant as indexed by AUC (bar plot).

Mean trajectories for conditions are presented in Figure 2. Positive AUC indicates when the mouse trajectory is above the idealized straight line, that is, when movement is attracted to the opposite category. The mean trajectory for Low frequency words showed attraction to the Nonlexical response button, and the mean trajectory for Pseudowords showed strong attraction to the alternative Lexical response.

To index *trajectory complexity*, values of AUC (a measure of spatial attraction toward the opposite response alternative) were analyzed with LMMs with two Random-effect factors (i.e., Subjects and Trials) and one Fixed-effect factor (Stimulus category). Mean AUC scores for Low Frequency words were higher than scores for High Frequency words (β _LowFreq_ = .2, t value = 3.5), and Pseudoword scores were higher than all other stimuli, in the order of Letters Strings (β _Pseudowords_ = .7, t value = 12), High Frequency words (β _Pseudowords_ = .5, t value = 8.2) and Low Frequency words (β _Pseudowords_ = .3, t value = 3.9). The negative coefficient for Letters Strings with respect to High Frequency words (β _LetterStrings_ = −.2, t value = −3.9) indicates that non-pronounceable stimuli were those less affected by competition. Low frequency words and Pseudowords were significantly more attracted to their own competing target category (i.e., Low Frequency words to non lexical stimuli and Pseudowords to lexical stimuli) than High Frequency words. This confirms that during the categorization process the stimuli with the most lexical ambiguity were affected by activation of competing categories. No difference emerged for Letter Strings, whose trajectories closely resembled the ideal straight response line.

#### Distributional analysis

**Figure 3:**
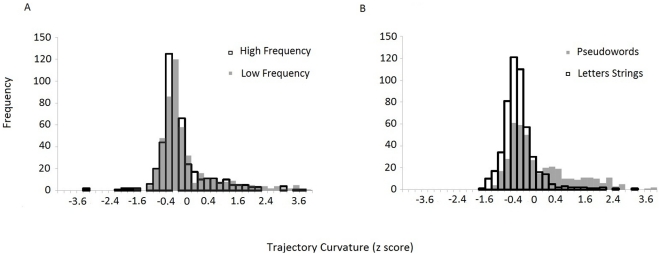
Distributions of trajectory curvature. a) Overlaid histograms of trajectory curvature for Lexical stimuli as measured by z scored values of AUC. High Frequency words exhibit unimodal distribution, whereas Low Frequency stimuli exhibit bimodality, with a first local maxima between a −.8 and −.4 z-score, and a second smaller mode between a .4 and .8 z-score. b) Overlaid histograms of trajectory curvature for Non Lexical stimuli as measured by z scored values of Area Under the Curve. Letters Strings have unimodal distribution. Pseudowords show bimodality with the first local maxima between a −.1 and −.4 z-score, and a second smaller mode between a .2 and .6 z-score.

Mouse Pseudoword trajectories showed attraction to the unselected response alternative. This effect might be due to the continuous attraction to the opposite category shown by all trials, or by a subpopulation of discrete-like errors, that is, cases in which movements were initially directed towards the unselected alternative followed by a sharp change in the response path towards the appropriate selected response. To test for the presence of this response subpopulation, we checked the distribution of trial-by trial AUC values for bimodality (see [22], [29]). The analysis was performed using Mouse Tracker to calculate the *bimodality coefficient (b)*. If *b* is greater than .555 the distribution in considered bimodal, if it is smaller it is considered unimodal. The AUC values for the different conditions were converted to z-scores within participants and then pooled across participants. Overall, the values of the four distributions show a sharp peak with negative skewness near the local maxima (see Figure 3).

The distribution of AUC values for High Frequency words (b = .48) and Letter Strings (b = .49) was within the unimodal zone, but distribution for Low Frequency words (b = .7) and Pseudowords (d = .58) exceeded the bimodality cut-off. Figure 3 shows double mode and negative skewness of the distributions, which appear less symmetrical for low frequency words and pseudowords, for which a subpopulation of response showed a drastic change in movement direction, indicating revision of an inappropriately selected response. The difference among distributions was further corroborated by the Kolmogorov-Smirnov test, which confirmed that distributions of high and low frequency words have statistically different shapes (p<.005), as do pseudowords and strings of letters (p<.005).

In sum, analysis of the within-category trials distribution shows that Low frequency words and Pseudowords were affected by competition from their alternative category, and that this competition did not continuously shape the curve of the trajectory but resulted in a sharp change of direction, consistent with a sudden revision process reflecting ongoing competition between partially active representations.

#### Trajectory time-course analysis

**Figure 4:**
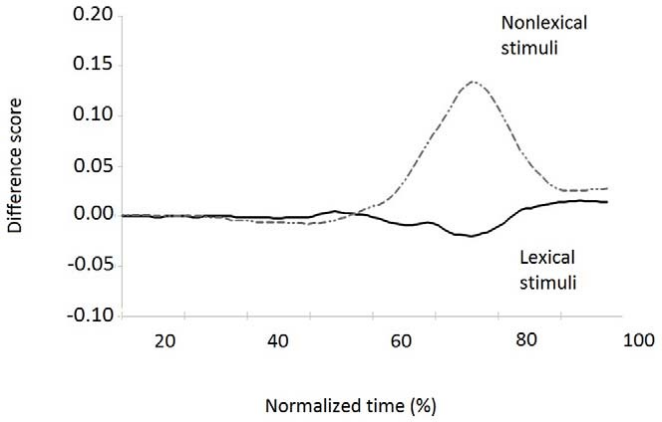
Intercategory difference score. Trajectory difference between Lexical and Nonlexical stimuli (dotted line). Averaged movement trajectories show difference between Pseudowords and Letter strings starting at the 43^rd^ normalized time (345 msec post-stimulus appearance), reaching the maximum amplitude at the 66^th^ time slice (690 msec post-stimulus appearance). Not much difference emerged for Lexical stimuli.

To characterize the spatial attraction, we computed a Difference score (Figure 4). Difference scores between “ambiguous" and “non ambiguous" items were computed separately for Lexical (Low frequency words – High Frequency words) and Non Lexical stimuli (Pseudowords – Letters Strings), and were plotted as a function of normalized time. This score index across time the degree to which the mouse traveled closer to the alternative category of ambiguous targets (i.e. Low Frequency words, Pseudowords) with respect to non-ambiguous targets (i.e., High Frequency words and Letters Strings).

As shown previously, the trajectory analysis confirmed that Lexical and Nonlexical stimuli induced different amounts of attraction to their opposite category.

For Lexical items, the Difference score was close to 0 over time. Although spatial attraction was present for Nonlexical items only, the effect was not continuous; initially, it was minimal with a substantial increase over time. LMMs was used to analyze difference scores in separate time windows: Time bin 1 (0–30), Time Bin 2 (31–60) and Time Bin 3 (61–90), with Stimulus Category (Lexical vs NonLexical) as fixed-factor and time bins as Random factor. Lexical category was automatically coded as the reference category. Differences emerged from the first time period (TB1: β _CatNonLex_ = −.001, p_MCMC_<.05), with the mean attraction score for NonLexical stimuli larger than the mean score for Lexical stimuli (TB2: β _CatNonLex_ = .02, p_MCMC_<.005; TB3: β _CatNonLex_ = .07, p_MCMC_<.005), indicating that nonlexical items were more attracted to the opposite category and that this effect increased over time.

## Discussion

**Figure 5:**
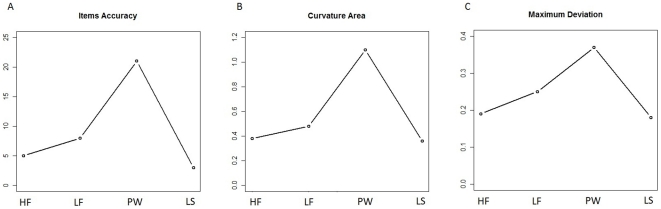
Line charts exhibit stimuli distribution along a “lexicality dimension". a) Percentage of Item Accuracy (categorization errors and Out of Time trials), increasing stimulus ambiguity resulted in higher percentage errors; b) Trajectories Curvature Areas, more pronounced for Pseudowords, which deviate from the ideal straight response line; c) Trajectories Maximum Deviation, more pronounced for Pseudowords, which deviates from the ideal straight response line.

The continuous recording of participants' mouse movements allowed us to unfold the process underlying the lexical decision task over time. We hypothesized that lexical stimuli are arranged along a “lexical dimension line", with some items with some items easy to distinguish because they have either rich lexical representations (high frequency words) or no representations at all (strings of letters), and other more ambiguous/difficult stimuli with weaker (low frequency words) or only partial representations (pseudowords). Our results support this hypothesis. Indeed, in several response measures the stimuli are organized in a u-shaped distribution of graded difficulty, as depicted in Figure 5.

Overall, lexical decision time and accuracy rate (which are the typical measures considered in this task) were significantly modulated by the type of stimuli to categorize, with Low frequency words and Pseudowords more difficult to process than the other stimuli, as indicated by lower accuracy and slower reaction times. Thus, lexical decision response time indicates that participants were more confident about their responses on High frequency words and unpronounceable non-words than on Low frequency words and Pseudowords. This finding is consistent with previous reports of expert readers' performance on lexical decision tasks in which the standard “button-press" procedure [30]–[32] was used and extends to kinematics measurements.

More interestingly, several measures of kinematics were significantly modulated by lexicality, thus revealing the underlying internal dynamics of the decision process. Analysis of the mouse movements showed that the Pseudoword trajectories were attracted to the lexical category. This attraction was not continuous; it was a sharp deviation from the initially selected direction. Thus, participants initially committed to the lexical (incorrect) response and then subsequently switched their commitment to the (correct) nonlexical response. Partial activation of orthographic representations initially points the decision toward the lexical category, and the subsequent top-down revision process from phonology and semantics (as completion of the orthographic processes) correctly drives towards the nonlexical category. For Low Frequency words, that is, items with weak lexical representations, averaged trajectory was smooth, with graded attraction to the alternative category. The averaged trajectories of the High Frequency words and Letter Strings showed direct pursuit of the correct response, with no efficient competitors.

Results are consistent with dynamic models of perceptual decision-making (in our case, visual word recognition), which emphasize the activation of multiple competing hypotheses and a graded nature of the response. One such model is Price and Devlin's [19] *Interactive Activation account*, which builds on predictive coding [10]–[13] to account for involvement of the ventral occipitotemporal (vOT) cortex in reading, and posits that activation of this region is modulated by the stimuli to be processed as a result of continuous interactions between bottom-up visual information and top-down predictions. This explains the widespread involvement of the left hemisphere's vOT in a range of tasks pertaining language processing, written [33] or auditory [34], but also to non-orthographic stimuli [35] and task context [36], which question its specificity for (written) language. In the lexicality dimension, pseudowords would increase their activation relative to letter strings because they resemble real words and engage top-down predictions from phonological areas. Moreover, pseudoword activation would also be greater than real word activation because they (pseudowords) elicit higher prediction errors, due to the poor match between predictions (generated by partially sharing phonological representations of real words) and the predicted visual representations. In the same vein, prediction errors should be higher for low frequency than high frequency words because the latter take advantage of the strong association between visual, phonological and semantic codes. Early involvement of mouth-articulatory regions in covert recognition of written language provides further support for this view [37], [38]. The mouse movements we recorded during the processing of pseudowords are consistent with an interactive activation account in which visual information (plausibly collected first) determines an initial bias towards the “lexical" hypothesis causing a low prediction error. This process is reinforced by the successful matching of phonological information (which causes a low prediction error). At a later stage of processing, when more information (e.g., semantic) becomes available, the high prediction error could determine a top-down revision and a rapid switch towards the “non-lexical" hypothesis. The differences in processing between Low and High Frequency words can be explained in terms of slower and quicker minimization of the prediction error.

Spivey's [8] attractor model also explains our results. If one considers the two responses (lexical vs. non-lexical) as two attractors, the lexical category can initially “pull" pseudowords and then “push" them when semantic information becomes available. Other dynamic models, such as drift-diffusion [6] and race models [7], need to be extended to explain our results. They were primarily developed to study choices that are completed at action onset. This is not the case in our set-up: Although the initial movement direction is informative, the ongoing choice can be revised at any time during the movement. A recent extension of accumulator models of decision-making permits dealing with such “changes of mind" and, at least in principle, can be adopted to explain our results [39]. Overall, our results provide support for dynamic models of decision-making. Nevertheless, further studies are necessary to decide between them and to test their specific hypotheses. For instance, predictive coding requires the contribution of two neural populations, one for encoding prediction (e.g., lexical stimulus prediction) and one for encoding prediction error (e.g., error in the lexical stimulus prediction). This hypothesis can be tested by by simultaneously measuring neural activity (e.g., in vOT) and manipulating prior information and expectation in the trials (see [40] for a similar approach in visual processing).

Although dynamic models of decision-making are popular in linguistic processing, for example, in spoken word recognition [14]–[17], it is commonly assumed that the source of dynamicity is external, for example, the fact that a spoken word unfolds over time. Here we applied the same logic, for the first time, to investigate visual lexical decisions. An important difference from previous studies is that in our set-up stimuli were short words that could be read with a single fixation [41]. This allowed us to avoid (or at least minimize) external sources of dynamicity, and to demonstrate that the internal processing of lexical decisions is also dynamic and competitive.

By using a continuous (kinematic) measure, we were able to unfold this dynamical internal processing over time. The use of an action-dynamics approach allowed us to visualize the link between written language processing and hand movements, with mouse trajectories mirroring online mental processing. Indeed, the results we obtained using this technique suggest a different view of mental processes, that is, as an integrative loop between perception, cognition, and action [42]). Whether the decision process regards perceptual discrimination [5], reaching decisions [4], processing language [9], categorizing faces [43] or perceiving different races [29], it does not proceed in an “all-or-nothing" fashion; rather, as it unfolds over time, it produces a cascaded formation of dynamic representations, which are largely shared across subsystems of perception and action.

## References

[1] Pagliuca G, Arduino LS, Barca L, Burani C (2008). Fully transparent orthography, yet lexical reading aloud: The lexicality effect in Italian.. Language and Cognitive Processes.

[2] Barca L, Bello A, Volterra V, Burani C (2010). Lexical-semantic reading in a shallow orthography: evidence from a girl with Williams Syndrome.. Reading and Writing.

[3] Barca L, Castrataro M, Rinaldi P, Caselli M (2011). Written language processing in hearing and deaf..

[4] Cisek P, Kalaska JF (2010). Neural Mechanisms for Interacting with a World Full of Action Choices.. Annu Rev Neurosci.

[5] Gold J, Shadlen M (2001). Neural computations that underlie decisions about sensory stimuli.. Trends in Cognitive Sciences.

[6] Ratcliff R (1978). A theory of memory retrieval.. Psychological Review.

[7] Usher M, McClelland JL (2001). On the time course of perceptual choice: The leaky competing accumulator model.. Psychological Review.

[8] Spivey J (2007). The continuity of mind. Oxford University Press, USA. Friston, K. (2005) A theory of cortical responses.. Philos Trans R Soc Lond B Biol Sci.

[9] Friston K (2005). A theory of cortical responses.. Philos Trans R Soc Lond B Biol Sci.

[10] Friston K (2010). The free-energy principle: a unified brain theory?. Nat Rev Neurosci.

[11] Friston KJ, Daunizeau J, Kilner J, Kiebel SJ (2010). Action and behavior: a free-energy formulation.. Biol Cybern.

[12] Rao RPN, Ballard DH (1999). Predictive coding in the visual cortex: A functional interpretation of some extra-classical receptive-field effects.. Nature Neuroscience.

[13] Gaskell MG, Marslen-Wilson WD (2002). Representation and competition in the perception of spoken words.. Cognitive Psychology.

[14] Magnuson JS, Tanenhaus MK, Aslin RN, Dahan D (2003). The time course of spoken word learning and recognition: Studies with artificial lexicons.. Journal of Experimental Psychology: General.

[15] McClelland JL, Rumelhart DE (1981). An interactive activation model of context effects in letter perception, Part 1: An account of basic findings.. Psychological Review.

[16] Revill KP, Aslin RN, Tanenhaus MK, Bavelier D (2008). Neural correlates of partial lexical activation.. Proc Natl Acad Sci USA.

[17] Shiffrin RM, Steyvers M (1997). A model for recognition memory: REM: Retrieving Effectively from Memory.. Psychon Bull Rev.

[18] Spivey M, Grosjean M, Knoblich G (2005). Continuous attraction toward phonological competitors.. Proceedings of the National Academy of Sciences of the USA.

[19] Price CJ, Devlin JT (2011). The Interactive Activation Account of ventral occipitotemporal contributions to reading.. Trends in Cognitve Science.

[20] Tsetsos K, Usher M, McClelland JL (2011). Testing multi-alternative decision models with non-stationary evidence.. Front Neurosci.

[21] Dehaene S, Changeux JP (2003). Development of elementary numerical abilities: a neuronal model.. Journal of Cognitive Neuroscience.

[22] Freeman JB, Ambady N (2010). Mousetracker: software for studying real-time mental processing using a computer mouse-tracking method.. Behavioral Research Methods.

[23] Barca L, Burani C, Arduino LS (2002). Word naming times and psycholinguistic norms for Italian nouns.. Behavior Research Methods, Instruments & Computers.

[24] Burani C, Barca L, Ellis AW (2006). Orthographic complexity and word naming in Italian: Some words are more transparent than others.. Psychonomic Bulletin & Review.

[25] Barca L, Ellis AW, Burani C (2007). Context-sensitive rules and word naming in Italian children.. Reading and Writing: An Interdisciplinary Journal.

[26] Baayen RH, Davidson DJ, Bates MD (2008). Mixed-effects modeling with crossed random effects for subjects and items.. Journal of Memory and Language.

[27] Brysbaert M (2007). ‘The language-as-fixed-effect fallacy’: Some simple SPSS solutions to a complex problem (Version 2.0)..

[28] Bates D, Maechler M (2009). lme4: Linear mixed-effects models using s4 classes [Computer software manual].. http://CRAN.R-project.org/package=lme4.

[29] Freeman JB, Pauker K, Apfelbaum EP, Ambady N (2010). Continuous dynamics in the real-time perception of race.. Journal of Experimental Social Psychology.

[30] Ellis AW, Ferreira R, Cathles-Hagan P, Holt K, Jarvis L, Barca L (2009). Word learning and the cerebral hemispheres: from serial to parallel processing of written words.. Philosophical Transaction Royal Society B.

[31] Kinoshita S, Lupker SJ, Rastle K (2004). Modulation of regularity and lexicality effects in reading aloud.. Memory and Cognition.

[32] Paulesu E, Mc Crory E, Fazio F, Menoncello L, Brunswick N (2000). A cultural effect on brain function.. Nature Neuroscience.

[33] Dehaene S, Cohen L (2011). The unique role of the visual word form area in reading.. Trends in Cognitive Sciences.

[34] Yoncheva YN, Zevin JD, Maurer U, McCandliss BD (2010). Auditory selective attention to speech modulates activity in the visual word form area.. Cerebral Cortex.

[35] Song Y, Hu S, Li X, Li W, Liu J (2010). The role of top-down task context in learning to perceive objects.. The Journal of Neuroscience.

[36] Twomey T, Duncan Kawabata KJ, Price CJ, Devlin JY (2011). Top-down modulation of ventral occipito-temporal responses during visual word recognition.. NeuroImage.

[37] Cornelissen P, Kringelbach ML, Ellis AW, Whitney C, Holliday IE (2009). Activation of the Left Inferior Frontal Gyrus in the First 200 ms of Reading: Evidence from Magnetoencephalography (MEG).. PlosONE.

[38] Barca L, Cornelissen P, Simpson M, Urooj U, Woods W (2011). The neural basis of the right visual field advantage in reading: A MEG analysis using virtual electrodes.. Brain & Language.

[39] Resulaj A, Kiani R, Wolpert DM, Shadlen MN (2009). Changes of mind in decision-making.. Nature.

[40] Egner T, Monti JM, Summerfield C (2010). Expectation and surprise determine neural population responses in the ventral visual stream.. Journal of Neuroscience.

[41] Blais C, Fiset D, Arguin M, Jolicoeur P, Bib D, Gosselin F (2009). Reading between eye saccades.. PlosOne.

[42] Pezzulo G, Barsalou L, Cangelosi A, Fischer M, McRae K (2011). The Mechanics of Embodiment: A Dialogue on Embodiment and Computational Modeling.. Frontiers in Cognition.

[43] Freeman JB, Ambady N (2011). Hand movements reveal the time-course of shape and pigmentation processing in face categorization.. Psychonomic Bulletin Review.

